# Spontaneous cortical MEG activity undergoes unique age- and sex-related changes during the transition to adolescence

**DOI:** 10.1016/j.neuroimage.2021.118552

**Published:** 2021-09-10

**Authors:** Lauren R. Ott, Samantha H. Penhale, Brittany K. Taylor, Brandon J. Lew, Yu-Ping Wang, Vince D. Calhoun, Julia M. Stephen, Tony W. Wilson

**Affiliations:** a Institute for Human Neuroscience, Boys Town National Research Hospital, Boys Town, NE, USA; b Department of Pharmacology & Experimental Neuroscience, University of Nebraska Medical Center, Omaha, NE, USA; c Department of Biomedical Engineering, Tulane University, New Orleans, LA, United; d Tri-institutional Center for Translational Research in Neuroimaging and Data Science (TReNDS), Georgia State University, Georgia Institute of Technology, Emory University, Atlanta, GA, USA; e Mind Research Network, Albuquerque, NM, USA

**Keywords:** Magnetoencephalography (MEG), Resting state, Frequency, Power, Development

## Abstract

**Background:**

While numerous studies have examined the developmental trajectory of task-based neural oscillations during childhood and adolescence, far less is known about the evolution of spontaneous cortical activity during this time period. Likewise, many studies have shown robust sex differences in task-based oscillations during this developmental period, but whether such sex differences extend to spontaneous activity is not understood.

**Methods:**

Herein, we examined spontaneous cortical activity in 111 typically-developing youth (ages 9–15 years; 55 male). Participants completed a resting state magnetoencephalographic (MEG) recording and a structural MRI. MEG data were source imaged and the power within five canonical frequency bands (delta, theta, alpha, beta, gamma) was computed. The resulting power spectral density maps were analyzed via vertex-wise ANCOVAs to identify spatially-specific effects of age, sex, and their interaction.

**Results:**

We found robust increases in power with age in all frequencies except delta, which decreased over time, with findings largely confined to frontal cortices. Sex effects were distributed across frontal and temporal regions; females tended to have greater delta and beta power, whereas males had greater alpha. Importantly, there was a significant age-by-sex interaction in theta power, such that males exhibited decreasing power with age while females showed increasing power with age in the bilateral superior temporal cortices.

**Discussion:**

These data suggest that the strength of spontaneous activity undergoes robust change during the transition from childhood to adolescence (i.e., puberty onset), with intriguing sex differences in some cortical areas. Future developmental studies should probe task-related oscillations and spontaneous activity in parallel.

## 1. Introduction

Neuroimaging of resting state brain activity is a rapidly growing field, with the vast majority of studies using fMRI to examine changes in blood oxygenation-level dependent (BOLD) signals when participants are relaxed and not engaged in tasks that probe specific cognitive functions (Azeez and Biswal, 2017; Biswal et al., 1995; Yang et al., 2020). However, there is growing interest in exploring other dimensions of resting state activity (e.g., spontaneous brain dynamics) using methods like magnetoencephalography (MEG). MEG is noninvasive, silent, and has excellent temporal (∼1 ms) and spatial precision (∼3–5 mm; Baillet, 2017; Wilson et al., 2016), making it ideal for examining spontaneous cortical activity in diverse populations such as developing youth (Hoshi and Shigihara, 2020). That said, relatively little is known about the development of resting state neural dynamics assessed by MEG, especially in children and adolescents.

Recent resting state MEG and electroencephalography (EEG) studies suggest that neural dynamics change with age (Gómez et al., 2013; Hunt et al., 2019; Meng and Xiang, 2016; Schäfer et al., 2014), although this work has generally examined adults and focused on connectivity among resting state networks (i.e., not changes in spontaneous cortical activity). The studies that have examined spontaneous regional activity suggest general age-related trends such as increased power in higher frequencies and decreases in lower frequency power with age (Gómez et al., 2013). More specifically, delta and theta power exhibit gradual decreases with age (Hoshi and Shigihara, 2020), while beta and gamma power typically increase with age (Gómez et al., 2013; Hunt et al., 2019). Using a large sample of 6–45 year-olds and MEG, Hunt et al. (2019) found that age-related changes in spontaneous activity were often regionally specific, such that alpha increased with age in the temporal lobe and gamma increased with age primarily in the frontal lobe (Hunt et al., 2019). While the number of MEG studies of spontaneous cortical activity in children is limited, there have been numerous studies using EEG and these suggest a shift from low frequency to high frequency power throughout development (Michels et al., 2013; Segalowitz et al., 2010; Uhlhaas et al., 2010). For instance, in a study of 8- to 12-year-olds during eyes closed rest, relative power of delta and theta decreased with increasing age, while alpha and beta relative power increased (Barry and Clarke, 2009).

EEG studies of resting state have also revealed sex differences throughout development, although this area has not been as extensively studied. For instance, Barry and Clarke (2009) found that females have higher relative delta, beta, and theta power, whereas males have higher relative alpha power during late childhood. One limitation of the existent EEG resting state studies is that most have reported electrode-level data (i.e., not performed source imaging), which is less spatially specific, making it difficult to decipher where in the brain these developmental, sometimes sexually-divergent, shifts in spontaneous dynamics are occurring. Identifying the cortical origin of these changes may provide critical information about the systems in play, which allows for a more thorough understanding of the trajectory of brain development. Such data is especially critical given the known complexities and intricacies of cognitive development during this sensitive period.

While the number of studies on functional development, and specifically development of spontaneous dynamics is limited, structural maturation has been extensively studied using MRI and it is likely that the trajectory of structural changes is predictive of developmental alterations in the neural dynamics. Essentially, throughout childhood and adolescence, the brain continually builds and refines neural connections through processes of synaptogenesis, synaptic pruning, and myelination, which is thought to help the brain to operate more efficiently (Khundrakpam et al., 2016; Selemon, 2013). These processes lead to gradual changes in brain structure, including cortical thickness and gray/white matter volume changes. These changes are especially robust during the onset of puberty (typically from 8 to 16 years old; Blakemore et al., 2010; Giedd et al., 1999; Sisk and Foster, 2004; Stephen et al., 2021). Specifically, early changes in global cortical gray matter follow an inverted U-shaped pattern, while white matter undergoes a linear increase through young adulthood (Casey et al., 2005; Giedd et al., 1999; Vijayakumar et al., 2016; Wierenga et al., 2014). That said, different regions of the brain follow distinct developmental trajectories and typically track the refinement of sensory, motor, and cognitive functions (Frangou et al., 2021; Gazula et al., 2021; Wierenga et al., 2014).

Sensory regions of the brain, including the somatosensory, auditory, and visual regions, structurally mature first, followed by temporal and frontal association regions (Gogtay et al., 2004; Wierenga et al., 2014). In addition to such age related changes, a higher level of variability in male cortical structure compared to females suggests that sex hormones may play an important role in brain development (Assari, 2020; Koolschijn and Crone, 2013; Taylor et al., 2020). Interestingly, many of the well-known changes in cortical structure appear to coincide with known shifts in spontaneous dynamics. For example, regions located more frontally in the brain reach adult levels of spontaneous power later than the posterior regions (Gasser et al., 1988b,a; Marek et al., 2018; Matousek and Petersén, 1973), a pattern similar to that observed structurally. However, far less is known about how spontaneous dynamics shift during the transition from childhood to adolescence. Given the robust age- and sex-related shifts in cortical structure (Faghiri et al., 2019, 2018; Mills and Tamnes, 2014), resting-state networks (Fair et al., 2008; Hunt et al., 2019; Stevens et al., 2009), and task-related brain oscillations (Embury et al., 2019; Killanin et al., 2020; Taylor et al., 2021, 2020a,b; Wiesman and Wilson, 2019) that occur during adolescence, it is likely that spontaneous neural dynamics will also show significant age and sex related changes throughout this transition. In the current study, we use MEG imaging to map changes in spontaneous cortical activity during the transition from childhood to adolescence. As reviewed above, previous resting state studies have largely focused on adulthood, with limited data examining spatially-specific age and sex differences in spontaneous activity in youth, despite abundant evidence that the brain undergoes key changes during this time period. Mapping the developmental trajectory of cortical physiological changes during the transition from childhood to adolescence is critical, as this is the temporal window when many psychopathological symptoms first emerge (e.g., anxiety and depressive disorders) and foundational data on normative development will enable markers of atypical physiology to be identified and used toward developing early interventions in future work. Therefore, our primary objective was to quantify the developmental shifts in spontaneous neural dynamics in a large sample of 111 healthy children and adolescents (56 females) ranging from 9 to 15 years of age. Our primary hypothesis was that both age and sex would have effects on spontaneous brain dynamics during development. More specifically, we predicted that the pattern of higher frequency power increasing with age and lower frequency power decreasing with age would follow a different developmental trajectory in males and females.

## 2. Methods

### 2.1. Participants

We enrolled 127 healthy adolescents (61 Male, 119 right-handed) who participated in the study (*M*_age_ = 11.78 years, *SD* = 1.60, range = 9.03–15.20 years; see Table 1). Exclusionary criteria included inability to complete the full resting scan, any medical illness affecting CNS function, neurological or psychiatric disorder, history of head trauma, current substance abuse, any medication known to affect CNS function, and the standard exclusion criteria related to MEG acquisition (e.g., dental braces, metal implants, battery operated implants, and/or any type of ferromagnetic implanted material). All demographic data were reported by a parent or legal guardian as part of the intake process. Parents of the child participants signed informed consent forms, and child participants signed assent forms before proceeding with the study. The Institutional Review Board at the University of Nebraska Medical Center reviewed and approved this study, and all protocols were in accordance with the Declaration of Helsinki.

### 2.2. MEG and MRI data acquisition

All MEG recordings took place in a one-layer magnetically-shielded room with active shielding engaged for environmental noise compensation. A 306-sensor Elekta/MEGIN MEG system (Helsinki, Finland), equipped with 204 planar gradiometers and 102 magnetometers, was used to sample neuromagnetic responses continuously at 1 kHz with an acquisition bandwidth of 0.1 – 330 Hz. The same instrument was used across all recordings. Participants were seated in a custom-made nonmagnetic chair, with their heads positioned within the sensor array, and were instructed to rest with their eyes closed for six minutes. Participants were monitored by a real-time audio-video feed from inside the shielded room throughout MEG data acquisition. Structural T1-weighted images were acquired on a Siemens 3T Skyra scanner with a 32-channel head coil and a MPRAGE sequence with the following parameters: TR=2400 ms; TE=1.94 ms; flip angle=8°; FOV=256 mm; slice thickness=1 mm (no gap); base resolution=256; 192 slices; voxel size=1 × 1 × 1 mm.

### 2.3. Structural MRI processing and MEG-MRI coregistration

Participants’ high-resolution T1-weighted MRI data were segmented using the standard voxel-based morphometry pipeline in the computational anatomy toolbox (CAT12 v12.7; Gaser and Dahnke, 2016) within SPM12. T1 images then underwent noise reduction using a spatially-adaptive non-local means denoising filter (Manjón et al., 2010) and a classical Markov Random Field approach (Rajapakse et al., 1997). An affine registration and a local intensity transformation were then applied to the bias corrected images. Finally, preprocessed images were segmented based on an adaptive maximum a posterior technique (Ashburner and Friston, 2005) and a partial volume estimation with a simplified mixed model of a maximum of two tissue types (Tohka et al., 2004). Images were normalized to MNI template space and the resulting segmented files were then imported into Brainstorm for coregistration.

Prior to MEG acquisition, four coils were attached to the participants’ heads and localized, together with the three fiducial points and scalp surface, using a 3-D digitizer (Fastrak 3SF0002, Polhemus Navigator Sciences, Colchester, VT, USA). Once the participant was positioned for MEG recording, an electrical current with a unique frequency label (e.g., 322 Hz) was fed to each of the coils. This induced a measurable magnetic field and allowed each coil to be localized in reference to the sensors throughout the recording session. Since coil locations were also known in head coordinates, all MEG measurements could be transformed into a common coordinate system. With this coordinate system (including the scalp surface points), each participant’s MEG data were co-registered with their structural MRI prior to source space analyses using Brainstorm.

### 2.4. MEG data pre-processing

Each MEG dataset was individually corrected for head motion and subjected to noise reduction using the signal space separation method with a temporal extension (tSSS; MaxFilter v2.2; correlation limit: 0.950; correlation window duration: 6 s; Taulu and Simola, 2006). MEG data processing was completed in Brainstorm (Tadel et al., 2019) and largely followed the analysis procedure outlined in (Niso et al., 2019). A high pass filter of 0.3 Hz and notch filters at 60 Hz and at its harmonics were applied. Cardiac artifacts and eye movements were identified in the raw MEG data and removed using an adaptive signal-space projection (SSP) approach, which was subsequently accounted for during source reconstruction (Ille et al., 2002; Uusitalo and Ilmoniemi, 1997). Data were then divided into four-second epochs and scanned for artifacts on a per-person basis. Specifically, epochs with amplitudes and/or gradients exceeding ±3 SD of that participant’s distribution of values were marked as bad and excluded from further analyses. Briefly, in MEG, the raw signal amplitude is strongly affected by the distance between the brain and the MEG sensor array, as the magnetic field strength falls off sharply as the distance from the current source (i.e., brain) increases. To account for this source of variance across participants, as well as other sources of variance, we used an individualized threshold based on the signal distribution for both amplitude and gradient to reject artifacts. The mean number of accepted epochs was 65.6 with a SD of 6.78 and a range of 50–89 epochs. The number of accepted epochs did not significantly vary as a function of age (*r*(111) = 0.10, *p* = .30) or sex (*t*(109) = −0.70, *p* = .48).

### 2.5. MEG source imaging and frequency power maps

As in (Niso et al., 2019), we computed minimum norm estimates normalized by a dynamic statistical parametric mapping (dSPM) algorithm for source imaging. To account for environmental noise, we utilized empty room recordings to compute a noise covariance matrix for source imaging (Baillet et al., 2001). The forward model was computed using an overlapping spheres head model (Huang et al., 1999). Finally, the imaging kernel of depth-weighted dSPM constrained to the individual cortical surface (Dale et al., 2000) was computed.

Using these source estimates, we then computed the power of cortical activity in canonical frequency bands: delta (2–4 Hz), theta (4–8 Hz), alpha (8–12 Hz), beta (15–30 Hz), and gamma (30–80 Hz). We used Welch’s method for estimating power spectrum densities (PSD; Welch, 1967) on each four-second epoch for each MEG recording, with one second sliding Hamming windows overlapping at 50%. We then standardized the PSD values at each frequency bin to the total power across the frequency spectrum. For each participant, we then averaged PSD maps across epochs to obtain one set of PSD maps per participant. Finally, we projected these maps onto the MNI ICBM152 brain template (Fonov et al., 2009) and applied a 3 mm FWHM smoothing kernel. Ultimately, it was these normalized source maps per frequency band that were used for further statistical analysis.

### 2.6. Statistical analyses

To further visualize the source power in each of the frequency bands, regions of interest (Brainstorm “scouts”) of the frontal, parietal, temporal, and occipital lobes were applied to each participant’s PSD map, and the average relative power across each lobe was extracted for each participant.

We then analyzed the whole-brain PSD maps in SPM12 to examine for spatially specific effects of age and sex and their interaction. For each frequency band, we ran an ANCOVA with age as a continuous predictor and sex as a categorical predictor and modeled the respective interaction term. To correct for multiple comparisons, we applied threshold free cluster enhancement (TFCE; Smith and Nichols, 2009) with a weighting factor of *E* = 0.6 and a cluster level family wise error (FWE) of 0.05 to the resulting statistical maps. Finally, F maps were thresholded by utilizing the clusters that survived correction. Data from peak vertices were used to display the corresponding effects.

## 3. Results

### 3.1. Descriptive statistics

Of the 127 healthy children who participated in the study, nine participants were missing MRI scans and seven were missing MEG data. Those participants were excluded from analysis. The results presented here include data from the remaining 111 participants (*M*_age_ = 11.89 years, *SD* = 1.60; range = 9.03–15.20). Demographic characteristics are detailed in Table 1.

### 3.2. Development of spontaneous neural dynamics

As stated above, we computed a series of ANCOVA models to identify the developmental trajectories of spontaneous neural dynamics. Specifically, we modeled the main effects of sex, age, and their interaction on spontaneous power within each of the six canonical frequency bands. Using TFCE and FWE 0.05 corrections, we were able to isolate clusters with significant effects, which we describe in detail below.

#### 3.2.1. Main effect of sex

**Fig. 1.**
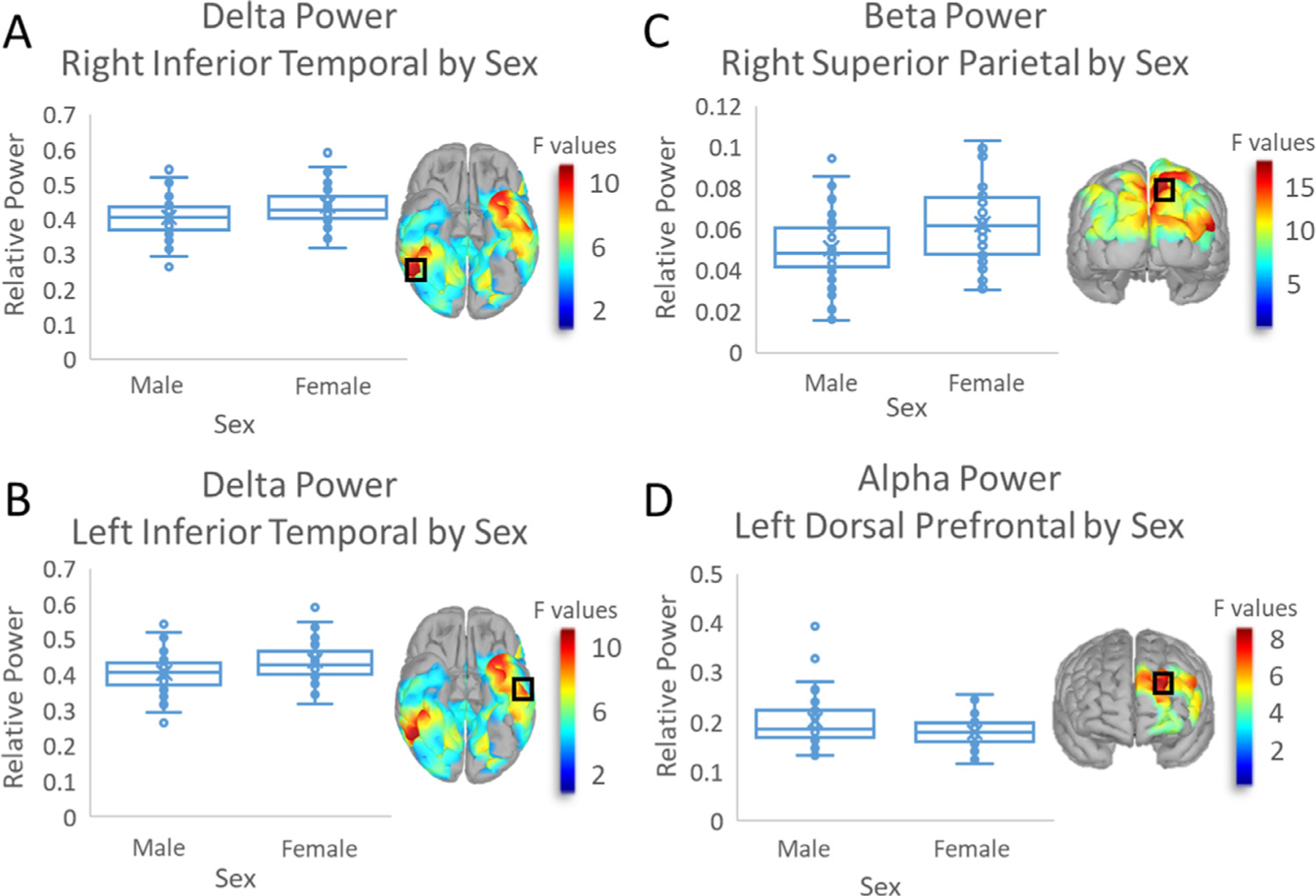
Main Effect of Sex: Box and whisker plots display significant sex effects by frequency band and cluster. Collapsing across age, plots show relative power on the *y*-axis and sex on the *x*-axis. F-maps thresholded with TFCE are superimposed on the corresponding plots, with a black box indicating the peak. The color bar to the right of each F-map displays the scale of respective *F* values. Delta power was higher in females than males in bilateral right (A) and left inferior temporal (B) clusters. Beta power in the right superior parietal was higher in females relative to males (C). Alpha power was higher in males than in females in the left dorsal prefrontal cluster (D).

Models of spontaneous power in the delta, alpha, and beta frequencies showed main effects of sex across multiple clusters (Fig. 1). Specifically, there was a main effect of sex in the delta range, where females had greater delta power than males in bilateral inferior temporal cortices (right: *F*(1107) = 10.292, *p*_*FWE*_ = 0.017; left: *F*(1107) = 9.357, *p*_*FWE*_ = 0.017; Fig. 1A-B). Females also exhibited significantly greater beta power compared to males across parieto-occipital regions, especially in the right superior parietal area (*F*(1107) = 15.81, *p*_*FWE*_ = 0.019; Fig. 1C). Conversely, males exhibited greater alpha power relative to females in left dorsal prefrontal cortex (*F*(1107) = 8.38, *p*_*FWE*_ = 0.035; Fig. 1D).There were no main effects of sex that survived TFCE correction in the theta or gamma frequency bands.

#### 3.2.2. Main effect of age

**Fig. 2.**
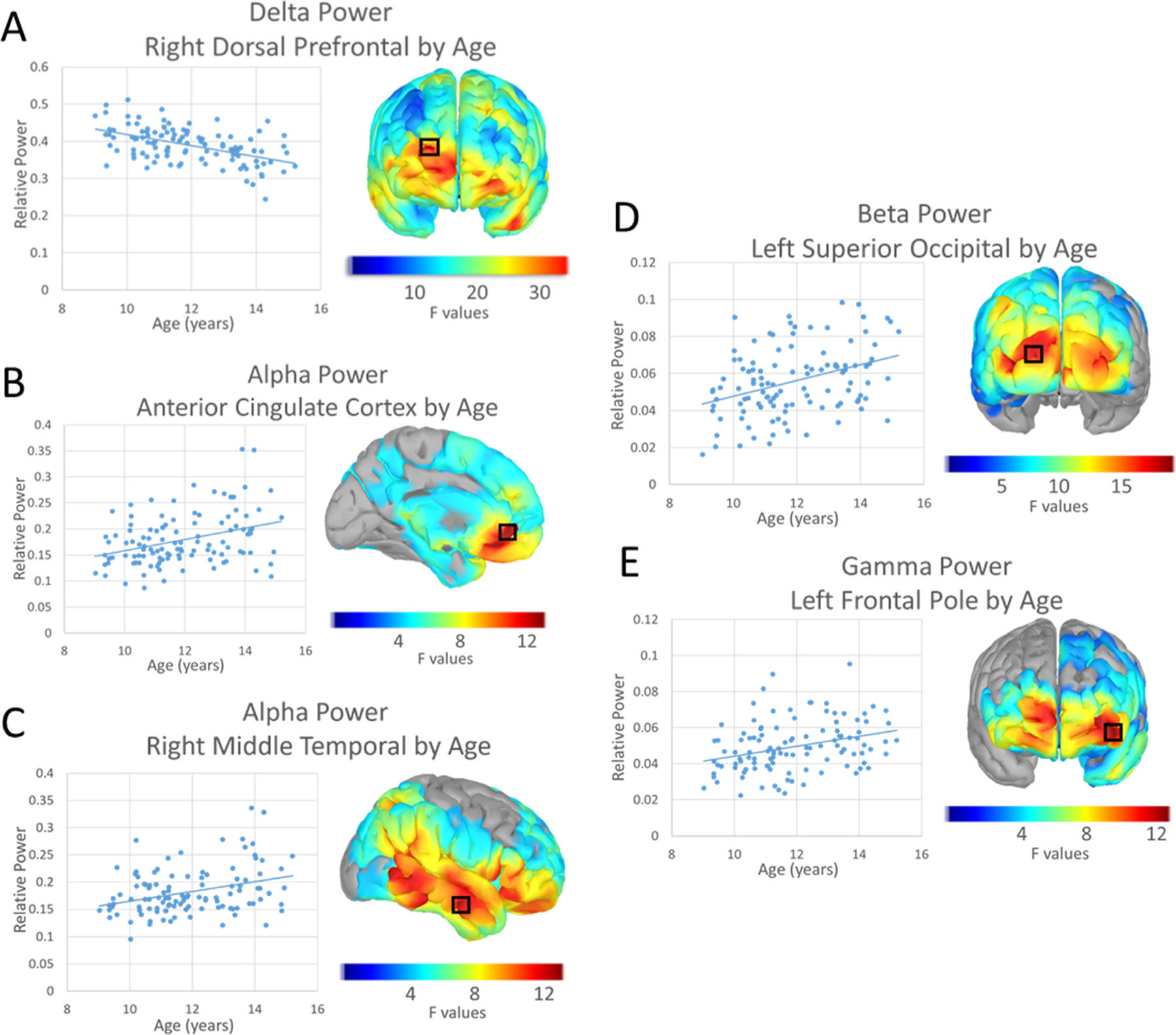
Main Effects of Age: Each participant’s relative power from the overall peak (maximum *F* value) is graphed in a scatterplot by frequency band and cluster. Relative power is shown on the *y*-axis and age on the *x*-axis. Trendlines are included on each graph indicating an increase or decrease in relative power with age. F-maps thresholded with TFCE are displayed with a black box indicating the peak. The color scale bar of *F* values is shown under each map. Delta power decreases with age in the right dorsal prefrontal cortex (A). Alpha power in the anterior cingulate cortex (B) and right middle temporal gyrus (C) increases with age. Beta power in the left superior occipital cortex (D) increases with age. Gamma (E) power increases with age in the left frontal pole.

Broadly, the data revealed decreases in delta power as a function of age, whereas power generally increased with age in alpha, beta, and gamma frequencies (Fig. 2). More specifically, delta power decreased with age across the whole brain with a peak located in the right dorsal prefrontal cortex (*F*(1107) = 36.015, *p*_*FWE*_ < 0.001; Fig. 2A). In contrast, alpha showed spatially specific increases in power spanning the frontal, temporal, and posterior parietal cortices, extending into the lateral occipital area and peaking in the anterior cingulate cortex (*F*(1107) = 13.202, *p*_*FWE*_ = 0.028) and the right middle temporal gyrus (*F*(1107) = 13.060, *p*_*FWE*_ = 0.014; Fig. 2B-C). Spontaneous beta power increased with age across the occipital, parietal, and left temporal cortices, with the strongest age effects in the left superior occipital area (*F*(1107) = 19.585, *p*_*FWE*_ = 0.006; Fig. 2D). Finally, increases in gamma *(F*(1107) = 12.303, *p*_*FWE*_ = 0.022); Fig. 2E) were observed in the left frontal pole. There were no significant main effects in the theta band.

#### 3.2.3. Age-by-sex interactions

**Fig. 3.**
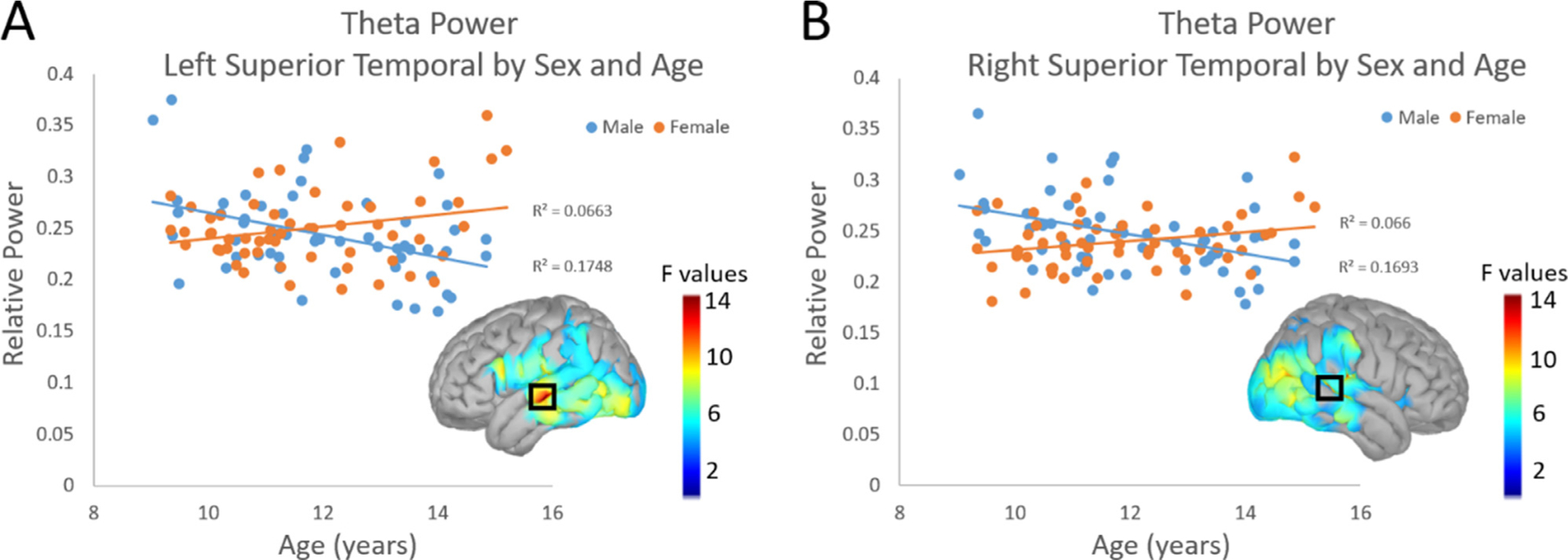
Interaction Effects of Sex and Age: Scatterplots display relative theta power on the y-axis and age on the *x*-axis in bilateral left (A) and right (B) superior temporal cortices. The blue circles and trendline in each plot represent males, while the orange circles and trendline represent females. In both regions, relative theta power decreased with age in males and increased with age in females. F-maps thresholded with TFCE are displayed with a black box indicating the peak. The color bar next to each map shows the scale of F values. Extracted values from the peak are plotted for each participant in the graph.

Finally, there was a significant interaction between age and sex in theta power (Fig. 3). In males, theta power decreased with age, whereas in females theta power increased with age. These effects were most robust in the left (*F*(1, 107) = 14.522, *p*_*FWE*_ = 0.025) and right (*F*(1, 107) = 13.815, *p*_*FWE*_ = 0.025) superior temporal cortices. No other interaction effects survived TFCE correction.

## 4. Discussion

In the current study, we examined the developmental trajectory of spontaneous cortical activity during childhood and adolescence. Specifically, we examined the extent to which spontaneous power in each of five canonical frequency bands was associated with age, sex, and their interaction in a large cohort of typically developing youth. We found several sex-specific effects across dorsal prefrontal and inferior occipitotemporal regions, including stronger spontaneous delta and beta in females relative to males, and stronger alpha power in males compared to females. With respect to age-related changes, delta power decreased with age broadly across the whole cortex, though the effects were strongest in dorsal prefrontal regions. Conversely, alpha, beta, and gamma, all increased in power with age across frontal, occipital, and temporal regions. Theta was the only frequency band to show an interaction between age and sex, with males showing decreased power in bilateral superior temporal cortices as a function of age, while females exhibited increased power with age in the same brain areas.

The sex differences we observed in resting state spontaneous activity are a novel finding that are not entirely surprising given the known sexually dimorphic patterns of functional and structural neural development. For example, numerous studies have shown sex differences in the task-based oscillatory dynamics underlying working memory (Embury et al., 2019), abstract reasoning (Taylor et al., 2020b), selective attention (Taylor et al., 2021), visual processing (Fung et al., 2021), and visuospatial attention (Killanin et al., 2020; Wiesman and Wilson, 2019) spanning theta, alpha, and gamma frequencies across spatially distributed areas. However, these findings are not universal; another study of motor processing in youth found no significant sex effects (Trevarrow et al., 2019), which may indicate that sex differences are more robust in high-level cognitive and sensory tasks. While less is known about sex-related differences in the spontaneous dynamics, our findings agree with the limited existing literature. Specifically, we found that females had higher delta and beta power, and males had higher alpha power regardless of age, which agrees with previous EEG work conducted at the scalp level (Barry and Clarke, 2009; Cragg et al., 2011). Thus, our findings extend this work by clarifying the cortical regions that underlie these sex-related differences in spontaneous cortical activity. In that regard, we found that regions of the bilateral inferior temporal gyri, commonly implicated in visual and memory processes (Han et al., 2002; Lin et al., 2020; Onitsuka et al., 2004; Su et al., 2014), exhibited the most robust sex differences in delta power (females > males), while a cluster in the left dorsal prefrontal cortex was critical to the sex differences observed for alpha (females < males). This brain region is commonly implicated in high-order cognitive functions such as decision making, planning, and language processing (Heekeren et al., 2006; Kaller et al., 2011; Klaus and Schutter, 2018). Lastly, compared to males, females exhibited stronger beta power, primarily in the right superior parietal lobe, which has been associated with spatial attention processes such as mental rotation (Coull and Frith, 1998; Harris and Miniussi, 2003; Harris et al., 2000; Szczepanski et al., 2010).

We also observed robust developmental changes in the spontaneous power within specific canonical frequency bands. Previous work has shown a shift from low to high frequencies with increasing age, with the power of delta and theta decreasing and that of alpha, beta, and gamma increasing at the whole brain level (Gómez et al., 2013; Hunt et al., 2019). This shift from lower to higher frequencies was also seen in our developmental sample, as the lowest frequency, delta, was found to decrease in power across development, while the higher frequencies (alpha, beta, and gamma) all increased with age. In addition to confirming prior work in this area, our data provides more granular information on the precise anatomical regions involved and focuses on a sensitive developmental transition period (i.e., puberty onset). The most prominent developmental effect was arguably in the delta range, which appeared to decrease in power with increasing age across the entire brain. This decrease was most robust in the right dorsal prefrontal cortex, which is a region that matures later than most brain regions, both structurally and functionally (Beveridge et al., 2014), is commonly associated with planning (Kaller et al., 2011), and has been linked to behavioral inhibition (Shackman et al., 2009). Contrary to delta’s prominent decrease in power, we found an increase in alpha power which extended across much of the cortex, with the strongest effects in the right middle temporal gyrus and the anterior cingulate cortex. Previous work has implicated the right middle temporal gyrus in the integration of visual and auditory processing streams (Binder et al., 2009; Papeo et al., 2019; Xu et al., 2019), while the anterior cingulate cortex has often been associated with executive control, specifically conflict monitoring and error detection (Botvinick et al., 2004; Carter et al., 1998). Given the data connecting alpha to attentional suppression (Foxe and Snyder, 2011; Klimesch, 2012), our findings suggest that this developmental period may be crucial to the maturation of attentional systems. Finally, gamma showed an increase in power in the frontal cortex, concentrating in the frontal pole. The frontal pole is poorly understood, but has been associated with cognitive function/processing and is one of the last areas in the brain to mature structurally (Bludau et al., 2014; Fuster, 2002; Romine and Reynolds, 2005; Tsujimoto et al., 2011). Likewise, the role of spontaneous gamma is poorly understood, although gamma range oscillations are known to emerge during childhood and continue changing through adulthood, playing a critical role in cognitive skill development (Gou et al., 2011; Tarullo et al., 2017). Future work should evaluate such changes in the context of gamma oscillations and determine the inter-relationships with cognitive function.

While our results replicate and extend previous age-related shifts from low (delta) to higher (alpha to gamma) frequency activity, we found a novel age by sex interaction in the theta range, which reflected a decrease in power with age in males and the opposite in females. Previous literature has found theta to decline in power with increasing age across the lifespan (Gómez et al., 2013; Hunt et al., 2019), but our data suggest that, at least during the transition from childhood to adolescence, this pattern may only be present in males with females exhibiting the opposite. This finding corroborates recent literature exploring the development of spontaneous neural dynamics. For instance, Hunt et al. (2019) noted a decrease in theta power with age, however there was notable heteroskedasticity specific to the younger participants in their sample suggesting unaccounted variance in their bivariate age-power correlations. It is plausible that this variance may have been due to sex-specific variability in developmental trajectories of children and adolescents that was not captured in their original analyses. Further supporting this argument, Hunt et al. (2019) demonstrated markedly sensitive shifts in theta-specific resting-state connectivity metrics as a function of age and sex, stating that the findings were, “potentially implicating that sexual dimorphisms mainly localize to this band” (p. 511).

This trajectory of sex-specific change in spontaneous theta may relate to the differing structural and functional maturational trajectories between males and females (Taylor et al., 2020, 2020b). For example, in an MEG study of abstract reasoning, Taylor et al. (2020b) found that theta oscillations in males increased with age in frontoparietal regions, while such theta responses decreased in females. The theta frequency band is often associated with a wide variety of cognitive functions, including memory processing, and long range network communication within the brain (Colgin, 2013; Herrmann et al., 2016; Mizuhara and Yamaguchi, 2007; Taylor et al., 2020b). While age and sex alone did not predict the trajectory of theta power, it is important to acknowledge that the interaction between age and sex was influential specifically to this frequency. In short, the known sex-related differences observed in brain structure throughout development likely influence theta activity within the brain, particularly regarding how network communication is occurring between regions with separate maturational trajectories.

Before closing, it is important to note limitations of the study, including its cross-sectional design. A longitudinal study would have provided more precise data and allowed stronger conclusions. In addition, the population used in this study reflected the demographics of the local community, and it’s possible that some of the findings may not fully generalize to cohorts with vastly different demographics. Another limitation is that we only examined linear age-related changes in the present study. Further investigations should expand on this line of work and determine whether there are non-linear patterns of age-related change in these dynamics among children and adolescents. Finally, pubertal development is another major factor to consider throughout this age range, and future studies should examine whether these sex and age specific findings are related to hormonal changes seen throughout development (e.g., (Fung et al., 2020)).

In summary, our study found age and sex dependent changes in spontaneous cortical activity from childhood to adolescence during an eyes-closed resting state MEG recording. Regarding sex, males exhibited higher alpha power compared to females, while females displayed higher delta and beta power compared to males. For age related changes, delta power was found to decrease with age, while alpha, beta, and gamma all increased in power with age. Theta showed an interaction between age and sex, whereby males had decreased power with age and females had increased power with age. Our findings suggest that spontaneous cortical dynamics may have a similar trajectory and even mirror structural cortical maturation throughout development. Many of these findings may directly relate to the cognitive changes seen with increasing age during the transition from childhood to adolescence, but future studies will need to further examine this possibility. By understanding these changes in spontaneous activity, we make steps forward in understanding the context of task-based changes in cortical oscillations of sensory, motor, and cognitive functions throughout development.

## Figures and Tables

**Table 1:** Demographic characteristics of the final sample.

	Male	Female	p-Value
Mean Age (years)	12.01	11.77	0.43
Age Range (years)	9.03–14.85	9.34–15.20	–
Race (White/Black or African American/Other/Unknown)	49/1/2/3	43/3/7/3	0.25
Ethnicity (Not Hispanic or Latino/Hispanic or Latino)	50/5	52/4	0.71
Handedness (R/L/both)	49/5/1	54/2/0	0.28

Note: Differences in age between males and females were assessed using an independent samples *t*-test; differences in race, ethnicity, and handedness were assessed using a chi-square test.
